# Necessity under construction – societal weighing rationality in the appraisal of health care technologies

**DOI:** 10.1017/S1744133120000341

**Published:** 2021-10

**Authors:** Tineke Kleinhout-Vliek, Antoinette de Bont, Bert Boer

**Affiliations:** Erasmus School of Health Policy & Management, Erasmus University Rotterdam, P.O. Box 1738, 3000 DR Rotterdam, the Netherlands

**Keywords:** deliberative decision-making, health care decision-making, necessary health care, priority setting, societal weighing

## Abstract

Health care coverage decisions may employ many different considerations, which are brought together across two phases. The assessment phase examines the available scientific evidence, such as the cost-effectiveness, of the technology. The appraisal then contextualises this evidence to arrive at an (advised) coverage decision, but little is known about how this is done.

In the Netherlands, the appraisal is set up to achieve a societal weighing and is the primary place where need- and solidarity-related (‘necessity’) argumentations are used. To elucidate how the Dutch appraisal committee ‘constructs necessity’, we analysed observations and recordings of two appraisal committee meetings at the National Health Care Institute, the corresponding documents (five), and interviews with committee members and policy makers (13 interviewees in 12 interviews), with attention to specific necessity argumentations.

The Dutch appraisal committee constructs necessity in four phases: (1) allowing explicit criteria to steer the process; (2) allowing patient (representative) contributions to challenge the process; (3) bringing new argumentations in from outside and weaving them together; and (4) formulating recommendations to societal stakeholders. We argue that in these ways, the appraisal committee achieves societal weighing rationality, as the committee actively uses argumentations from society and embeds the decision outcome in society.

## 1. Introduction

Whether a health care technology is made available to patients, for example through a (national) formulary, benefits package, or insurance scheme, is decided by means of health care coverage decisions. Making these decisions well is notoriously complex as it leans on a wide variety of heterogeneous considerations that are brought together in the decision-making process (Vuorenkoski *et al*., 2008; Hughes and Doheny, 2011; Cerri *et al*., 2014; Russell and Greenhalgh, 2014; Dakin *et al*., 2015). The most-studied part of this process is the assessment, which examines the available scientific evidence such as the (cost-) effectiveness of the technology, often employing Health Technology Assessment (HTA) methods (e.g. Le Polain *et al*., 2010; Franken *et al*., 2012; Kleijnen *et al*., 2012; Salas-Vega *et al*., 2016; Makady *et al*., 2017). Notably, such scientific knowledge bases have been shown essential but not sufficient for making good coverage decisions (Moes *et al*., 2016; Calnan *et al*., 2017; Samenleving, 2017; Shirazi *et al*., 2017). Therefore, many coverage decisions feature a second part, sometimes (but not always) a distinct step in time and space: the appraisal. An appraisal entails the formulation of a (recommended) coverage decision based on contextualisation of the given evidence (Oliver *et al*., 2004; Walley, 2007; Patera and Wild, 2014; Jansen *et al*., 2017; Kleinhout-Vliek *et al*., 2020). Previously, contextualisation has broadly been defined as taking into account a variety of values and considerations (Patera and Wild, 2014). Some, such as the English National Institute for Health and Care Excellence (NICE), has specified contextualisation to mean establishing ‘what is good for society’, or in other words, providing a societal weighing of the evidence (Culyer and Rawlins, 2004; NICE, 2008; Shah *et al*., 2013).

How societal weighing works, and how exactly appraisal committees may achieve this, remains underexplored. Recent descriptive work on deliberative coverage decisions more generally highlights that these decisions display *rationality* different from formalised, institutionalised rationalities generally prescribed for such decisions (Jenkings and Barber, 2004; Russell and Greenhalgh, 2014; Russell, 2017). We derive three distinct elements of this type of rationality from the literature. First, this type of rationality utilises different knowledge types. These types include not only the scientific input deriving from the assessment but often also knowledge provided by patients as well as ‘local’ knowledge regarding the institutional setting where the decision-making process takes place. Second, constructing this type of rationality involves being able to respond appropriately, humanely, to these different types of input and combine them into one decision, and deal effectively with any tensions that may arise between knowledge types (Jenkings and Barber, 2004; Gkeredakis *et al*., 2011; Hughes and Doheny, 2011; Moreira, 2011; Russell and Greenhalgh, 2014; Moes *et al*., 2016; Calnan *et al*., 2017). Third, the process of construction of this type of rationality adheres to substantive requirements, which are generally in the form of formalised decision criteria as well as processual requirements (Russell, 2017), often those laid out by the widely-used Accountability for Reasonableness framework (Daniels and Sabin, 1997, 1998, 2008; Daniels, 2000; Hasman and Holm, 2005). These three elements would appear, however, to be described for coverage decisions generally, so the question remains: what does societal weighing rationality look like specifically?

In this study, we will fill the gap concerning societal weighing rationality by examining the appraisal phase of Dutch health care coverage decisions, which like NICE's decisions described above, explicitly aim for a ‘societal weighing’ of the evidence base provided in the assessment (Zorginstituut Nederland, 2016, 2017). Furthermore, Dutch decision-making practice provides an excellent case because the appraisal is set up as a separate meeting, a distinct moment in time and space, making it easier to distinguish from the assessment phase (Commissie-Dunning, 1991; Stolk *et al*., 2001, 2002). In the Netherlands, the National Health Care Institute (in this text: ‘the Institute’, in Dutch: *Zorginstituut Nederland*), an arm's length body, is responsible for advising the Ministry of Health on the contents of the basic benefits basket. This basket outlines the bare-minimum health care insurance package that is obligatory for all Dutch citizens. The Dutch coverage decision-making process usually starts with a scoping session, in which stakeholders are invited to contribute relevant considerations. This is followed by a meeting of the Scientific Advisory Committee (‘the assessment committee’, in Dutch: *Wetenschappelijke Adviesraad*), in which the scientific knowledge base is established based on HTA methodology (Franken *et al*., 2012; Kleijnen *et al*., 2012; Salas-Vega *et al*., 2016; Makady *et al*., 2017). The Package Advisory Committee (‘the appraisal committee’, in Dutch: *Adviescommissie Pakket*), convened once-monthly at the Institute, is subsequently responsible for the societal weighing of the evidence and the formulation of coverage advice to the Minister (Couwenbergh *et al*., 2013; Zorginstituut Nederland, 2016, 2017). The Institute uses four formalised criteria in this advice, namely effectiveness, cost-effectiveness, feasibility (including budget impact considerations), and necessity. ‘Necessity’ is, as a consequence of being a formalised criterion, present in the documents providing input for the appraisal as they outline the available information per criterion. It has, however, also been considered to be established especially in appraisal (Mastenbroek *et al*., 2006; Couwenbergh *et al*., 2013; Kleinhout-Vliek *et al*., 2017; Zorginstituut Nederland, 2017; Kleinhout-Vliek *et al*., 2020). Therefore, this paper will explore how the appraisal committee *constructs necessity*, as this may aid in uncovering elements that are specific to the achievement of societal weighing rationality (Poley *et al*., 2002; Stolk *et al*., 2002; Hoedemaekers and Oortwijn, 2003).

### 1.1 Aim

This paper describes the phases of constructing necessity by examining the contents of and the proceedings at Dutch appraisal meetings through observations, transcriptions, and subsequent analysis of audio recordings of two meetings. These meetings varied greatly in terms of the type of health care technology appraised. This is supplemented with interviews with appraisal committee members and Institute employees (*n* = 13) and observations of other appraisal meetings (*n* = 4). We answer the question: how does the Dutch appraisal committee construct necessity?

## 2. Methodology

### 2.1 Approach

In order to explore the Dutch appraisal, we analyse two cases of specific coverage decisions. Argumentations used to construct necessity that could potentially be employed by the appraisal committee include, but are not limited to, the morbidity and need experienced by the patient, availability of alternative treatments, the financial cost per individual patient, and compassion with vulnerable groups such as children or small numbers of patients generally. These necessity argumentations are part of a list of 20 argumentation types derived from a realist review of argumentations used to establish the necessity of coverage of health care technologies worldwide (Kleinhout-Vliek *et al*., 2017). For an overview of these 20 inductively-formulated argumentation types, see Table 1. The Netherlands is a particularly fruitful setting for studying the construction of necessity as the Dutch use two of these 20 necessity argumentations, namely individual severity of illness (argumentation type ‘Morbidity/Severity’) (Franken *et al*., 2015; Reckers-Droog *et al*., 2018, 2019) and the cost that the individual patient will incur (argumentation type ‘Individual Cost’), as their explicit, *a priori* formulated necessity criterion. As such, these two argumentations ought to be present in every decision document inputting into the appraisal (Stolk *et al*., 2001; Hoedemaekers and Oortwijn, 2003; Couwenbergh *et al*., 2013; Niëns, 2014). Necessity argumentations are of interest for two reasons. First, these argumentations are employed not only by professional decision-makers but also by other parties, such as patients and other societal stakeholders, who may also be present at the appraisal meeting (further information below). Second, necessity argumentations are variable in usage as their perceived validity and allotted weight differs per decision, making their patterns especially vivacious (Kleinhout-Vliek *et al*., 2017; Kleinhout-Vliek *et al*., 2020).

**Table 1.** Overview of the 20 argumentation types that fall under the necessity criterion and their respective descriptions (Kleinhout-Vliek *et al*., 2017)

Argumentation type	Description
Definition of Illness	Whether the ailment is considered an illness for which treatment is necessary
Dignity	Whether (lack of) coverage is considered to affect the dignity of the patient to such an extent that it needs to be amended
Equity/Fairness/Justice	Whether coverage would be necessary to counter injustice/inequity/lack of fairness in (access to) treatment
Human Right	Whether (lack of) coverage is considered to affect the human rights of the patient to such an extent that it needs to be amended
Individual Cost	Whether lack of coverage would stop patients from buying necessary care themselves due to prohibitive cost
Individual Responsibility	Whether the individual is considered responsible for paying for this treatment
Medical Necessity	Whether or not a treatment is considered to be ‘medically necessary’ or a ‘medical necessity’
Morbidity/Severity	Whether the physical and/or psychosocial morbidity associated with a certain ailment constitutes such a need that coverage is considered necessary
Moral Hazard	Whether there is considered to be a possibility of over-usage (i.e., unnecessary increase in demand, when people use more than they need as a result of coverage)
Need	The extent to which the patient is considered to be in need for which treatment is necessary
(No) Alternative	Whether or not viable alternatives are considered to be present which would make coverage more or less necessary
Patient-Diagnosis	Whether an illness is self-reported rather than diagnosed by a doctor
Range of Normality	Whether the experience of the patient is considered normal or abnormal to such an extent that coverage is deemed necessary
Rule of Rescue	Whether the identifiability of individuals close to death is considered to heighten the necessity of coverage
Similar Treatments	Whether similar treatments are covered or not (meaning that this type of treatment is considered necessary)
Small Number of Patients	Whether the small size of the patient population is considered to heighten the necessity of coverage (due to, amongst others, the inequality in terms of research expenditure or difficulties in obtaining high-quality data)
Societal Impact	Whether coverage is considered necessary to allay the impact this disease has on people beyond the patient
Societal Functioning	Whether coverage would aid a person's necessary functioning in society
Societal Responsibility	Whether society is considered responsible for paying for this treatment
Vulnerability/Compassion	Whether a compassionate response to vulnerable groups, e.g. children, in the form of coverage is considered to be a necessity

To study necessity argumentations in appraisal we have chosen a case approach (Ragin, 2004; Creswell and Poth, 2017) because researching a similar process in a variety of situations is considered helpful for gaining insight into commonalities across situations, especially when it concerns context-dependent social processes (Lamont, 2012; Dussauge *et al*., 2015). In this, we hold that the context is case-specific and thus differs per decision situation (Asdal, 2012; Asdal and Moser, 2012). The cases chosen concern two health care technologies, namely eculizumab (Soliris^®^) and front teeth replacement therapy. These cases are relatively run-of-the-mill; only eculizumab was sparingly discussed in the Dutch media (Berkhout, 2017). They share two characteristics that resulted in their selection. First, the cases vary widely in terms of the type of technology, price, and a number of patients affected, but with necessity argumentations playing a pivotal role in both, as suggested in informal conversations by contacts at the Institute (front teeth replacement therapy) or the literature review [eculizumab (Soliris^®^)] (Kleinhout-Vliek *et al*., 2017). Second, the appraisal meetings took place in 2015 and 2016, meaning that the first author could be present at the appraisal meeting (see Table 2).

**Table 2.** Overview of data collected pertaining to the case studies

Case	Observations and audio files	Documents	Interviews
Eculizumab (Soliris^®^)	File 1 (October 2016)	1.1 discussion document	–
		1.2 appraisal report	
		1.3 patient contribution	
Front teeth replacement	File 2 (February 2015)	2.1 discussion document	Institute employee 3 (April 2015)
		2.2 appraisal report	Institute employee 4 (May 2015)

### 2.2 Methods

Our dataset comprises two cases. The data on these cases were gathered through observations, transcription of audio files, documents, and interviews. The Institute consented to the first author accessing the setting of the appraisal committee through an explicit invitation by the secretary of the committee. This invitation included the ‘closed’ pre-meeting for the eculizumab case. The secretary also provided audio-recordings s/he used to write minutes to be analysed for this study. The first author was present at both appraisal committee meetings, where she observed and took field notes. These notes were supplemented by audio files of the same appraisal committee meetings, which were transcribed verbatim. Moreover, we analysed five documents pertaining to these cases. For both cases, this entailed the document that was provided to the appraisal committee (the ‘discussion document’) and the final ‘appraisal report’ supplemented by the patient contribution for eculizumab (see Table 2). For the documents, only the main body of text was analysed (i.e., excluding appendices).

For triangulation purposes, the first author interviewed seven policy advisers (‘Institute employees’), some of whom worked on the cases. She also interviewed six appraisal committee members present at the meetings, some of whom were interviewed multiple times, to a total of 13 people over 12 interviews (for a precise overview of who was interviewed when, please see Table 2). All approached interviewees consented to be interviewed, except one Institute employee, who declined due to a full schedule. Three interviews were group interviews (type: field-formal, meaning that the questions were of a semi-structured nature and that the interviewer took on a somewhat directive role) (Frey and Fontana, 1991). Institute employees 2 & 5 and 1 & 7 were interviewed in pairs at the request of the employees themselves, as they considered their answers would supplement one another (in the former case, the interviewer did not know two people would be present until the moment of the interview). The group interview with committee members 1, 4, 5 & 6 was done out of convenience, as committee members are often only present at the Institute once a month, so this presented a good opportunity. Again, the interviewees considered their answers to complement one another. Oral informed consent was given for use of interview data, written informed consent was given for publication, and a formal waiver for ethical approval was obtained from the Medical Ethics Committee (in Dutch: Medisch Ethische Toetsings Commissie) at the Erasmus Medical Centre, Rotterdam, the Netherlands (reference number: MEC-2017-539. The interviews were conducted by the first author using a topic list with non-structured, open-ended questions, and the interviews were audio-recorded and subsequently transcribed verbatim. The topic list included for Institute employees: how this technology arrived at the Institute agenda, the scoping session, how they retrieved any additional information, the appraisal meeting, how they arrived at the text in the different documents, and more general topics pertaining to the Institute. This was done to gain deeper insight into the working processes at the Institute, especially concerning different forms of input for the appraisal meeting. For committee members, the topic list concerned the appraisal committee's functioning generally, how different types of information are usually dealt with, and specific experiences they could recall. These questions were formulated to gain insight into the cases, but also to obtain reflections on tentatively formulated phases of necessity construction. Lastly, all interviewees were probed about necessity argumentations.

This dataset was analysed as follows. The list of 20 necessity argumentations (Kleinhout-Vliek *et al*., 2017) was used as sensitising concepts to guide the first step of the detailed content analysis of the documents and transcribed committee meetings, in which the necessity argumentations were used as a list of codes (Table 1). The explicit mentioning, ‘black on white’ or ‘out loud’, of necessity argumentations was tracked across the documents and audio files through coding specific utterances as one or more of the argumentation types, using Microsoft Excel to put utterances that had received the same code together in the same row. The first tentative patterns were subsequently elucidated based on this tracked argumentation use. For the formulation of the phases, we chose a chronological and person-dependent (first this person spoke, then that person contributed) rather than substantive (these argumentations were used more often than those) patterning. These patterns formed the basis for the different phases, formulated and refined in further extensive discussions within the authorship team, then supplied and solidified by information retrieved from the interviews. These interviews specifically clarified the dynamics around the Institute employees' and the patient and patient representatives' contributions. Additional observations served to see whether data saturation was achieved (Table 3). A member check with the appraisal committee and several personal communications (committee meetings of October and December 2016 and February 2017, the audio file of a committee meeting of January 2015, member check through a presentation to the committee on 14 April 2018, personal communications with committee members 5, 6, and 7) to see whether the interpretation made by the authors stayed close enough to the interpretation of those observed (Sayer, 2011) (see also Table 3). Especially the member check and the personal communications have positively impacted the reliability of the study in this regard; the personal communications followed the member check to clarify the interpretation of our data in a number of places, these are indicated in the text through reference to ‘(interviewee X, personal communications)’.

**Table 3.** Overview of additional data collected

Committee meetings Observations and audio files	Interviews
January 2015 *Audio file only*	Committee member 5 (March 2015)
October 2016	Institute employee 6 (March 2015)
December 2016 *Observations only*	Committee member 3 (August 2015)
February 2017 *Observations only*	Committee member 2 (September 2015)
	Committee member 5 (October 2016)
	Institute employees 2 & 5 (October 2016)
	Institute employees 1 & 7 (October 2016)
	Committee member 1 (February 2017)
	Committee members 1, 4, 5 & 6 (February 2017)
	Committee member 6 (October 2017)

## 3. Results

This section first describes the working procedure of the Institute and the appraisal committee, succeeded by an introduction to the case studies and a description of the general setting, and finally descriptions of the way in which necessity is constructed per phase of the appraisal committee meeting.

### 3.1 Working procedure

The Institute's working procedure for formulating an advised decision follows the general assessment-appraisal pattern (Patera and Wild, 2014). Agenda setting varies and may happen through pharmaceuticals gaining market access or by another party, such as the Minister of Health or a professional organisation. Once it is placed on the agenda, one or two Institute employees take responsibility for this dossier; in this study, these were different people for each case. After a scoping session with interested parties, the scientific evidence reports are written by other Institute employees with expertise in therapeutic value, cost-effectiveness, and budget impact. These are bundled and combined with a short explainer by the one or two Institute employees who hold final responsibility for this dossier to benefit the assessment phase. The assessment phase takes place at Scientific Advisory Committee, the assessment committee in this text (in Dutch: *Wetenschappelijke Adviesraad*, WAR), based on which an assessment report is composed by the secretary of this committee. This report provides a summative conclusion on the valuation, the size, and the probability of the effect of the medicine. This assessment report is sent to the stakeholders for a consultation and consequently combined with input from the scoping session as well as a fresh explainer into a ‘discussion document’ by those responsible for this dossier. This is aided by the secretary of the assessment committee and approved by the secretary and chair of the appraisal committee, to benefit the appraisal phase (committee member 5, personal communications) (Zorginstituut Nederland, 2017).

The appraisal subsequently takes place at the meeting of the Package Advisory Committee (in this text: the appraisal committee). The committee is comparatively small (Patera and Wild, 2014): it comprises eight to ten external experts (e.g. in medical ethics, pharmaco-economics, or medicine), who are not employed by the Institute. Like NICE's Social Value Judgements, the Dutch appraisal explicitly aims for a societal weighing of the provided scientific evidence (Couwenbergh *et al*., 2013; Zorginstituut Nederland, 2016, 2017).

The appraisal committee members read the assessment report in advance of the meetings. All meetings, which are in principle open to the public, are preceded by a ‘closed’ meeting, in which patients and their representatives were absent but the Institute employee(s) responsible for the dossier were present, and the files are already discussed (observations February, November 2015, October, December 2016, and February 2017, see also Zorginstituut Nederland, 2016).

### 3.2 Cases

We studied the appraisal deliberations for two significantly varying cases, eculizumab and front teeth replacement therapy.

Eculizumab (Soliris^®^) is an orphan drug currently licensed for Atypical Hemolytic Uremic Syndrome (aHUS) and Paroxysmal Nocturnal Hemoglobinuria (PNH). After four years of provisional coverage, which is a temporary coverage arrangement, the final advised decision to the Minister was to be drafted by the Institute in 2016. The discussion document (document 1.1) states that there is a debate on whether the incremental cost-effectiveness ratio (ICER) for treatment of aHUS with eculizumab approximates the reference value at €80,000 per QALY for severe diseases. The calculated cost-effectiveness ratio was thus considered highly unfavourable, but clinicians and patient organisations had initiated independent research on shortening the treatment period through improved start-stop criteria, which was expected to result in a more favourable ICER. A grant was already obtained for this research (though not for actual medicine). After the formal presentation by the Institute employee responsible for the file came the contributions of one patient and two patient representatives (in this case, the mother of a patient and a clinician). Especially the mother's emotional contribution was followed by an extended silence on the part of the committee, and many committee members vocalised their appreciation of these contributions. For the deliberations, the research on the new protocol was the primary focal point. The committee thought investing in this a worthy cause; the final advised decision stressed that the committee considered the initiative so commendable that it needed to remain possible to reimburse eculizumab from public funds within the research protocol (document 1.2). Relief on the part of the patients was palpable; the chair suggested the committee take a break, and the patient (representative)s were congratulating each other, also a few committee members offered their congratulations (observations/audio file 1).

Front teeth replacement was discussed in the appraisal committee in February 2015 after the College of Dentists (in Dutch: *College van Adviserend Tandartsen*) placed it on the agenda through contact with Institute employees 3 and 4. The reason provided was that current legislation was perceived as a perverse stimulus, the situation being as follows. All dental care is covered by the Dutch basic benefit package until the insured's 18th birthday, but not afterwards. This means that when young people lose their front teeth or were born without them, they may prefer to have them replaced before their 18th birthday (as the costs are approximately €3500 for front teeth implants), whereas it is often better to do so later as the oral cavity is not fully grown until the age of 22. In the appraisal committee meeting where the coverage of front teeth implants was discussed, the topic was not considered of major importance or interest, even a little laughable, for its small budget impact (observations/audio file 2, interview with Institute employee 5). Institute employees 3 and 4 were especially aware of its political history; one regarded it as a mistake that could have been prevented that current legislation did not specify the extended coverage until the age of 22. The discussions in the appraisal committee seemed relatively straightforward, with everybody leaning in the direction of extending coverage until one committee member apparently wanted to stimulate the discussion by deliberately going against the tide (paraphrase, interview Institute employee 3). This resulted in a longer discussion, with the final decision apparently taken for pragmatic reasons (namely the time it would take to change the legislation; coming back to it in the appraisal meeting next month would mean another year's extension) (observations/audio file 2). The final advised decision, then, was that as long as the claim was made before the 18th birthday, coverage would be continued until the age of 22 (document 2.2).

### 3.3 General setting

The meetings of the appraisal committee take place in a sizeable meeting room that is relatively light, even though the blinds are drawn. There are two entrances: you can enter the room from ‘within’ the building (behind the security gates) but also from the ‘outside’, provided your name is on the list, which is checked at the reception. Ten people are seated around tables set up in a large square. There are thermoses with coffee and tea and plates of biscuits. An ‘audience’ of eight more people, including me, sits on the rows of chairs set up on one side of the room, where we can see the committee and the presentation screen well. I seem unable to shake the feeling we are watching a staged performance. It is clearly one of these occasions where you feel conscious of making noise: I open my water bottle as quietly as possible. Given this fairly formal setting, I am struck every time by how warmly the committee members greet one another when they come in, how at-home they seem (one even brought her dog!), and even more by the apparent light-heartedness of it all, the sheer good humour that characterises the proceedings. (Condensed field notes, observations 1 and 2)

In this setting, the deliberations on the two cases followed approximately the same order. We have separated this order out into four phases, namely (1) the contribution of the Institute employee(s); (2) the contributions of the patient(s) and/or their representative(s); (3) the actual deliberations of the committee; and (4) the formulation of the decision. This separation into four phases allows us to show how necessity is constructed in each phase.

### 3.4 Phase 1: institute employee(s)

The contribution of the Institute employee(s) is the first of four phases we distinguish in the Dutch appraisal meeting, and we will show the impact of these contributions on the committee deliberations that followed.

In our observations, after the meeting was formally opened by the chair the Institute employee was invited to summarise the assessment report by means of a presentation. This presentation contained information on the individual severity of illness (code: Morbidity/Severity, the codes correspond to argumentation types in Table 1) and costs per the individual patient (code: Individual Cost). These two are formulated as official, explicit elements of the formalised necessity criterion (Couwenbergh *et al*., 2013). Notably, the appraisal committee subsequently took the individual severity of illness and costs per individual patient as ‘given’ in their deliberation that followed; we noted they did not explicitly mention them (observations/audio files 1 and 2). As a committee member explained afterwards, the reason for this lack of use of the severity of illness and costs for the individual by the committee is that it is not their mandate to weigh these explicitly (committee member 4, personal communications). This does not mean the Institute employee's input is considered ineffective. In an interview, one committee member commented on the introduction by Institute employee(s) that:

CM5 [T]he discussion, at some point, heads into a different direction.

CM6 Yes, that's right. (Group interview with committee members 1, 4, 5 and 6)

They acknowledged the impact on the deliberation: the Institute employee's argumentations deriving from formalised criteria are considered authoritative and steering the direction the committee's discussion takes.

In sum, necessity is constructed by the appraisal committee not by weighing the formalised necessity criteria contributed by the Institute employee explicitly, but instead by allowing them to steer the appraisal process implicitly, which committee members consider to positively impact the deliberations.

### 3.5 Phase 2: patient (representative)(s)

For the second phase of the appraisal, which consists of the contribution(s) of the patient (representative)(s), we will also describe how they affect the deliberations of the committee.

During the appraisal meeting for the eculizumab case, we observed how patients and/or patient representatives, in this case, a mother and a medical doctor, gave a short statement following the Institute employee (observations/audio file 1). Interestingly, the necessity argumentations that patient (representative)s contributed were not mentioned by the appraisal committee. For example, the patient representative mentioned that the decision-making process was:

[A] story (…) that concerns (…) justice. (Document 1.3 and observations/audio file 1, code: Equity/Fairness/Justice)

Not only is [coverage] the best option for the doctors and us; it is also for society as a whole. (Document 1.3 and observations/audio file 1, code: Societal Impact)

We observed that neither of these argumentation types was mentioned by the appraisal committee (observations/audio file 1). The patient representative also brought in two argumentation types that were repeated once but not discussed further. First:

As of [29 October 2014], the life of our little daughter Rosa, just 1 year old, forever lost its ease and was never again taken for granted. (Document 1.3 and observations/audio file 1, code: Vulnerability/Compassion)

This clear call for compassion with eculizumab patients, and Rosa, in particular, was repeated by committee member 7 (observations/audio file 1), but not discussed by the committee. Thus, the committee did not explicitly weigh the patient (representative)(s)’ contributions.

Notably, however, patient (representative)(s)’ contributions were a primary topic of discussion during the interviews with committee members. Several committee members commented on what they experience when ‘faced with’ patients and their representatives during the appraisal:

We need to keep the distance [from the patients]. [With emphasis:] Someone needs to keep the distance. And it should be us. (…) It's like a war, the generals have to decide where the bombs will fall, and they should not see the mess it creates. (Committee member 2, interview)

[A good decision] requires a kind of distance from that specific [patient perspective]. (Committee member 3, interview)

To function well, the committee members feel they require metaphorical *distance* from the patients, which helps explain the lack of explicit discussion of argumentations contributed by patients or their representatives. Another committee member reflected in personal communication on the topic:

The patients challenge the committee to keep their position. (…) You need to stay detached. [But the patients’ input] gives handles for substantiating [your position]: you must explain it well. It challenges you as a group and as a person. (Committee member 7, personal communication)

This committee member suggested that the patients' input increases the quality of the final (advised) decision and its substantiation as the process is ‘challenged’ by the contributions.

Summarising, necessity is constructed by the committee during the deliberations by not weighing the patient (representative) contributions explicitly, but by allowing them to challenge the decision-making process implicitly, which committee members consider heightens the quality of the justification or rationale for the decision.

### 3.6 Phase 3: deliberation

The next phase we describe is the deliberative discussion by the committee, where many different argumentations are contributed by the committee.

In our observations, the deliberative discussion was initiated by the chair, with ample opportunity for the other committee members to speak and to respond to one another. The professed goal of the discussion is to ascertain whether there may be reasons to deviate from the reference value for cost-effectiveness (committee member 5, personal communications), which range in three classes from 10 to 80,000 euro per Quality-adjusted Life Year (QALY). If the cost-effectiveness falls within a certain reference value range given a certain individual severity of illness, it is classed as favourable; if it does not, it is classed as unfavourable (Zorginstituut Nederland, 2017, 2018). We observed how the discussions both started and ended with a ‘round’ around the meeting table, where committee members were invited to speak in turn. In the deliberative phase of the meeting, members make statements and respond to one another before the final decision (observations/audio files 1 and 2). The deliberations were subsequently summarised by the chair of the committee.

In interviews, both committee members and Institute employees describe the appraisal as an ‘open, moral’ place ‘with permeable borders’ where many ‘things’ interact ‘organically’ to form an advised decision (committee members 2, 6, 4, Institute employee 4, (group) interviews). Specifically, the observational data show that necessity is constructed during the deliberations by *bringing new argumentations together*. In the front teeth case, one appraisal committee member asked whether front teeth replacement therapy was potentially a form of cosmetic surgery, which would decrease the necessity of coverage significantly (as the absence of front teeth would be seen as relatively normal and the therapy thus as a cosmetic intervention; code: Range of Normality). In response, other committee members highlighted the importance of making sure young people are able to do normal things like eating an apple (audio file 2, code: Societal Functioning). This tension was resolved by one committee member, who humorously brought the following new necessity argumentations together:

I had a strange association with the contraception debate, where we said, "You should pay for that yourself”, but up to a certain age, we think that it needs to be reimbursed because of the situation that, just, for example, a 14, 15-year-old with parents who think otherwise would not be able to – that it could result in unwanted pregnancies, and we would like 18-year-old girls to enter adulthood without an unwanted pregnancy. [Laughter] They must both have good teeth and not have an unwanted pregnancy! [Laughter] (Committee member 9, observations/audio file 2, codes: Similar Treatments and Vulnerability/Compassion)

From this quote, we see how this committee member brought in new argumentations through comparison with contraceptives, which is covered in the basic benefits package for under-18s (and thus heightening the necessity of coverage; code: Similar Treatments). S/he also brings it together with argumentation of compassion with a 14, 15-year old whose parents might not be able to afford this therapy (code: Vulnerability/Compassion). This was confirmed in an interview, where a committee member described the general process as follows: the appraisal committee ‘brings in’ new necessity argumentations ‘from the outside’ to be ‘woven together’ (committee member 1, interview). In fact, this bringing in from the outside is part of their official task (art. 14, Zorginstituut Nederland, 2016). Sources of such new argumentations from the outside also include newspapers (as observed during committee deliberations, audio file committee meeting January 2015).

In sum, necessity is constructed in this phase by bringing in new argumentations derived from the outside, from society, with sources including newspapers and previously-taken decisions, and weaving these considerations together.

### 3.7 Phase 4: decision

In the final phase of the appraisal, the committee formulates its positive or negative coverage decision advice.

In this phase, we observed how the committee not only formulated the decision but also gave additional recommendations, generally phrasing their coverage advice as: ‘yes, provided that…’ or ‘no, unless…’ (observations/audio files 1 and 2). From our observations, it became clear that the research on the protocols outlining new start-stop criteria was crucial in the deliberations on eculizumab's coverage status and the final decision. The committee spent a lot of time outlining the tension between the high price and corresponding cost-effectiveness ratio and the risks the patients were willing to take by stopping treatment with eculizumab for a shorter or longer period under these new, to be researched further, protocols (observations/audio file 1). In the end, the advised decision to the Minister of Health was positive, with the strong recommendation that the research on the protocols containing the start-stop criteria would continue (document 1.2). Furthermore, we observed that the positive decision including recommendations was supplemented by committee member 13. S/he recommended that the eculizumab expertise centre responsible for the research would communicate the desire to pressurise the manufacturer for better pricing of eculizumab to other countries (observations/audio file 1).

The committee thus gives recommendations to a broad set of societal stakeholders including the manufacturer, the professional organisations involved in research, in addition to the Minister of Health. We analyse this dynamic as a way of completing the construction of necessity: with these recommendations, the decision is embedded in society as it is linked directly not just to patients and the Minister of Health, but to other societal stakeholders who will impact what care entails in practice.

## 4. Discussion

To describe how necessity is constructed in Dutch health care coverage decisions, we followed the use of necessity argumentations across meetings of the appraisal committee at the Dutch Health Care Institute, supplemented by document analysis and interviews. Necessity is constructed by the appraisal committee by first, allowing explicit criteria contributed by the Institute employee to steer the process; and second, by allowing patient (representative) contributions to challenge the process. The third element of necessity construction we identify is bringing in new argumentations from the outside, from society, and weaving them together carefully. Fourth and finally, necessity is constructed by the appraisal committee through formulating recommendations to societal stakeholders, thus making the decision more societally embedded than a tersely formulated ‘yes’ or ‘no’.

In the introduction, we outlined our interest in societal weighing rationality and concluded that achieving rationality in health care coverage decisions generally comprises (1) understanding different types of knowledge and (2) combining them into one decision, whilst (3) adhering not only to substantive requirements, but also processual ones (Jenkings and Barber, 2004; Gkeredakis *et al*., 2011; Hughes and Doheny, 2011; Russell and Greenhalgh, 2014; Calnan *et al*., 2017; Russell, 2017). In terms of societal weighing rationality specifically, we conclude that all three elements are confirmed by our dataset on constructing necessity to a certain extent. The committee meeting indeed features different types of knowledge. In this dataset, these contain on the one hand argumentations representing scientific knowledge contributed by the Institute employee (individual cost and severity of illness), and on the other hand, also the patient (representative)'s experiential knowledge. We show that although these considerations are generally not mentioned explicitly by the committee, they are considered to steer the discussions implicitly and to heighten the quality of the deliberations and the justification or rationale, respectively. Our dataset shows that during the deliberations, the committee does combine many argumentations, thereby also confirming the second element of rationality, and would appear to do so according to substantive and processual requirements laid down in policy documentation as per the third identified element (Couwenbergh *et al*., 2013; Zorginstituut Nederland, 2017), though the latter was not an explicit topic of study. This overlap with previously-described coverage decision rationality, we argue, indicates that these parts of constructing necessity may indeed be classified as elements of not only health care coverage rationality, but societal weighing rationality. This overlap between necessity construction and societal weighing rationality is even more evident for the latter two phases of constructing necessity, as they show how argumentations are brought in from society (in our dataset, sources included previous decisions but also newspapers), and how the (advised) decision is embedded in society (through recommendations to societal stakeholders) in turn. These two elements give a distinct flavour to societal weighing rationality that other studies of health care coverage decision making appear not to have hit upon to date.

The reluctance in terms of explicitly weighing the experiences of individual patients has previously been described for a variety of settings (Carlsen and Norheim, 2005; Rooshenas *et al*., 2015; Hashem *et al*., 2018). One potential underlying reason may be what Moreira describes as ‘the politics of singularities’. Personal stories, according to Moreira, have a strong allegorical character by which they may spark the imagination through being relatable, and are thus able to destabilise other argumentations (Moreira, 2012). This fact that the committee listens to but does not explicitly mention these argumentations may be a manifestation of a refusal to be drawn into such politics. Regardless of the underlying reason, this finding is fascinating in light of widespread attempts to draw patients and citizens into such decision-making processes (Wait and Nolte, 2006; Mitton *et al*., 2009), also termed a ‘multi-stakeholder appraisal’ (Abrishami *et al*., 2017). Our data underline that this will not be easily achieved, which is in line with earlier work on the practices of dealing with different types of knowledge in health care coverage settings (Moes *et al*., 2016; Hashem *et al*., 2018).

On the recommendations specifically, the brunt of the available literature covers the process of coming to these decisions and the rationales behind them, rather than looking at what the additional recommendations might be (cf., Giacomini *et al*., 2000; Singer *et al*., 2000; Martin *et al*., 2001; Madden *et al*., 2005; Bukachi *et al*., 2014; Byskov *et al*., 2014; Rooshenas *et al*., 2015). Follow-up research may address questions on whether other appraisal committees also give recommendations, on the underlying dynamic these recommendations may point to, the role these recommendations play, and their implications for the process of health care coverage decisions generally.

### 4.1 Strengths and limitations

This paper describes how a coverage appraisal is performed by decision-makers and the dynamics of using argumentations therein. To our knowledge, this study adds to existing research on coverage decisions both methodologically, through showcasing how insight can be generated by tracing argumentation types across documents and deliberative settings, as well as content-wise, noting what societal weighing rationality entails specifically. It contributes to the literature of elegant muddling through by showing the emergent shared systematics behind it (Calnan *et al*., 2017; Russell, 2017). Moreover, it shows how pragmatic rationality is accomplished collectively; it is not just the committee, but also the Institute employees and the patients that crucially shape the deliberations.

The methodology chosen will have impacted the data; the dataset comprises a mixture of nine individual and three group interviews. Though both interview types were held primarily for purposes of data triangulation, the data gathered in these settings will have differed. In group interviews, the members of the group may stimulate each other (rather than the researcher being the only one to take this role) in terms of encouraging recall, opinion elaboration, and variation in response. However, group members may also correct each other and even sway each other's opinions. Influential herein are group size, familiarity, and power dynamics (Frey and Fontana, 1991; King *et al*., 2018). In this dataset, two of the three group interviews were with two direct Institute employee colleagues, who seemed high on familiarity and relatively low on power dynamics, positively impacting the data gathered. Interviewees did indeed often supplement each other; both double interviews were, in fact, suggested by the interviewees themselves for that reason. The third group interview, with four appraisal committee members, also concerned peers who were comfortable expressing their opinions together. Moreover, in this case, the ‘quieter’ respondents were interviewed separately as well.

A major limitation of this study is the focus on two cases. It is relatively common to only focus on one case for characterising these types of decision making (Moreira, 2011; Moes *et al*., 2016). The case approach has granted us increased reliability but may necessarily lack some in-depth acquaintance with each case. Another limitation is the focus on the deliberations in the appraisal committee meeting only. Other studies focus on the ‘back stage’, thereby uncovering more work that is done ‘behind the scenes’ to accomplish these types of deliberations (e.g., Escobar, 2015). Future research could attempt to visualise both, especially elucidating how the two intermingle in practice (cf. Hajer, 2005).

## 5. Conclusion

Using heterogeneous argumentations to make well-justified decisions is a task that many public institutions work hard to complete astutely. This paper gives insight into the processes of tackling this task in a particularly vibrant field: health care coverage. It does so through examining the construction of necessity in the deliberative appraisal of two Dutch coverage decisions by following the necessity argumentations as mentioned by the different parties involved, supplemented by interviews with both appraisal committee members and Institute employees. Necessity is constructed differently in the four phases of the appraisal meeting, which, we show, correspond to four elements of societal weighing rationality. These elements comprise first, allowing explicit criteria to steer the deliberations implicitly. Second, being shaped by the input of patient (representative)s; these are considered to challenge the process and heighten the quality of the justification or rationale. Third, bringing in new argumentations from society and weaving them together, and fourth, formulating recommendations to societal stakeholders to place the decision ‘back’ into society. These latter two elements of societal weighing rationality, in particular, explicate how the committee reaches a decision that is well-embedded in society.
